# Extending standardized speech analysis across languages: An Italian toolkit for transcription and coding of neurological speech

**DOI:** 10.3758/s13428-026-03175-x

**Published:** 2026-09-14

**Authors:** Allegra Benzini, Alyssa M. Lanzi, Cinzia Palmirotta, Simona Aresta, Petronilla Battista, Gaia C. Santi

**Affiliations:** 1 Laboratory of Neuropsychology of Bari Institute, Istituti Clinici Scientifici Maugeri IRCCS, Bari, Italy; 2 Department of Communication Sciences & Disorders, University of Delaware, Newark, DE USA; 3 Delaware Center for Cognitive Aging Research, University of Delaware, Newark, DE USA; 4 Neurorehabilitation Unit of Lumezzane Institute, Istituti Clinici Scientifici Maugeri IRCCS, Brescia, Italy

**Keywords:** Speech and language annotation, Cross-linguistic, Neurological patients, TalkBank, Language adaptation

## Abstract

Speech and language analysis plays a central role in assessing adult neurological disorders, providing sensitive markers across clinical populations to support diagnosis. Standardized transcription and coding systems are essential to ensure reproducible analyses and enable comparisons across studies and languages. However, practical guidance on adapting such systems for non-English speakers remains limited. This work presents a structured toolkit for adapting speech and language transcription and coding conventions of adult speakers with neurological disorders from English to Italian. The adaptation builds on the CHAT conventions developed within the TalkBank framework. It follows the guiding principles of clarity, readability, and efficiency of data entry, while ensuring linguistic appropriateness for Italian. Original commands and coding options were systematically evaluated and then retained, modified, excluded, or added as needed, with the majority of changes in the coding process reflecting Italian-specific phonetic-phonological and morphosyntactic norms and typical impairment of aphasic production. Beyond providing a ready-to-use Italian manual, this toolkit defines a transparent and replicable framework for cross-linguistic adaptation. The manual was designed as a flexible resource in which users can select coding options guided by specific goals, allowing them to focus on relevant linguistic features at the word or sentence level or across different linguistic domains. By providing linguistically grounded conventions compatible with the TalkBank framework, the Italian Toolkit supports clinically meaningful speech analysis in Italian-speaking adults, and it offers a methodological model for extending standardized annotation systems to other languages.

**Supplementary Information:**

The online version contains supplementary material available at https://doi.org/10.3758/s13428-026-03175-x.

## Introduction

Language deficits commonly occur following acquired brain injury, particularly after vascular or traumatic lesions affecting the dominant left hemisphere (Ilie et al., 2017; Wilson & Schneck, 2021) and also appear in various neurodegenerative disorders. In some cases, these deficits emerge early as a specific and prominent symptom, as seen in primary progressive aphasia (PPA) (Mesulam et al., 2003; Gorno-Tempini et al., 2011), or they develop alongside broader cognitive impairments, as in Alzheimer’s disease (AD) (Caro et al., 2025). Notably, several studies have also highlighted their relevance as early markers in other disorders in which language impairment is not considered a core clinical feature (i.e., behavioral variant of frontotemporal dementia, Parkinson’s disease) (Aresta et al., 2025; García et al., 2022; Geraudie et al., 2021; Palmirotta et al., 2024). Linguistic markers reflecting specific changes in language use and production have therefore emerged as promising non-invasive and cost-effective tools for the early detection of these conditions (Alsheikhidris, 2024; Themistocleous & Stark, 2026). The analysis of extended speech production through connected or spontaneous speech tasks represents a valuable approach for examining language production beyond the single-word level, providing detailed information across multiple linguistic domains, including phonetic, phonological, lexico-semantic, morphosyntactic, syntactic, and discourse-pragmatic levels. Connected speech is an ecologically valid way to measure language output, but its transcription and analysis require tools that ensure methodological consistency, reproducibility, and detailed linguistic annotation.

The Codes for the Human Analysis of Transcripts (CHAT) format and the Computerized Language ANalysis (CLAN) software, both developed within the CHILDES/TalkBank framework (MacWhinney, 2000), provide a standardized and widely adopted approach for systematically transcribing and quantitatively analyzing connected speech. These computational tools enhance transcription accuracy, facilitate the sharing of transcript data, and enable the extraction of objective measures of language performance (MacWhinney, 2019). To preserve the readability of the transcriptions for both human and computational audiences, the CHAT/CLAN system meets three main requirements: (i) clarity, which requires that each symbol must have a well-defined and consistent real-world referent and that coding conventions must be applied systematically; (ii) readability, so transcripts should remain accessible and easy for human readers to process; (iii) ease of data entry, as CLAN provides automated checks, morphological and syntactic analyses, and semi-automatic coding tools to support transcribers, while leaving transcription itself primarily a human task.

Although originally developed for the analysis of child language data, CLAN has also proven particularly useful for studying language characteristics and impairments in adults with acquired neurological disorders. The TalkBank infrastructure now includes a wide range of open-access datasets involving adult speakers (Lanzi et al., 2023; MacWhinney & Fromm, 2022).

A substantial body of work has long shown that aphasia and language impairments manifest differently across languages, as a function of cross-linguistic structural variation. Seminal cross-linguistic research, such as Menn et al. (1989, work on agrammatic aphasia, demonstrated that language-specific grammatical and lexical structures critically shape the manifestation of aphasic symptoms, and subsequent studies have confirmed that language impairments in neurological conditions exhibit language-specific patterns rather than uniform manifestations, reflecting differences in morphological complexity, syntactic structure, lexical choices, and discourse strategies. Nevertheless, aphasiology and language-disorders research more broadly have remained strongly biased toward English (Beveridge & Bak, 2011), despite the fact that many impairment-relevant phenomena are highly language-dependent. Hence, there is a need for language-specific benchmarks and adapted assessment tools, as an English-centric approach can lead to diagnostic inaccuracies and disparities across research and clinical settings (Coppieters et al., 2024; García et al., 2023; Mazzeo et al., 2024; Santi et al. 2026).

Both the CHAT transcription system and CLAN analysis were originally developed in English for English-speaking Children. Thus far, those systems have been implemented in 28 different languages, and the database now contains over 44 million spoken words. For several languages, including Italian, all corpora have been tagged to automatically provide information on grammatical structures. One significant effort in adaptation has been done for the Mandarin language (see: https://talkbank.org/0info/manuals/Clin-CLAN-zho.pdf). Despite these important advancements, the application of CHAT and CLAN to Italian, particularly in adult neurological populations, still poses some challenges. Certain linguistically and clinically relevant features of Italian, as well as specific characteristics of neurological speech disorders (e.g., articulatory deficits), are not yet systematically captured within the current framework. In addition, the development of a formalized guide for CHAT/CLAN application may improve clinical practice efficiency.

In this paper, we describe the development of the Italian CHAT Toolkit, a structured extension that maintains the fundamental principles of CHAT/CLAN while carefully selecting or adding coding symbols to ensure consistency with the Italian language and relevance to the adult clinical population. Our aim is to provide clinicians with a more accessible approach to characterize speech and language impairments, while also offering a methodological framework to guide future language-specific adaptations for different populations of interest. Importantly, this study does not set out to provide a comprehensive cross-linguistic comparison between English and Italian; rather, it highlights the linguistic peculiarities of Italian and raises awareness that coding and analytical conventions developed primarily for English cannot always be directly applied. Moreover, the present work focuses on transcription and error-coding conventions within the standard CLAN workflow: it does not modify the morphosyntactic annotation layer (the %mor and %pos tiers), and existing Italian TalkBank resources for morphosyntactic analysis remain available and can be used alongside it.

## Methods

### Symbols inclusion and exclusion criteria

CHAT is the standard transcription and coding system for the TalkBank project. CHAT includes both basic and advanced formats for transcription and coding. MinCHAT is the most basic format required for CLAN commands to run successfully. There are also a variety of advanced transcription and coding options to increase precision. Examples of advanced options include a system for coding speech errors and a system for marking tone units, pauses, and contours. The basic units of CHAT transcription are morphemes, words, and utterances. Words constitute the fundamental building blocks of sentential and discourse structures; their analysis provides critical insights into the development and organization of syntax, morphology, discourse, and conceptual representations. Utterances, particularly in adult speech, are typically defined as units containing at least one main clause, generally including a subject and a main verb. In combination with CLAN, CHAT supports the systematic extraction of multi-level linguistic features across large corpora. Analyses can be conducted at the level of utterances (e.g., number, length, and grammatical and semantic well-formedness), lexical items (e.g., token frequency and distribution across grammatical classes), and error patterns. The framework allows for the structured annotation of errors across different language domains (e.g., phonological, lexical, morphosyntactic) and enables the automatic computation of their frequencies across multiple transcripts, facilitating large-scale, reproducible quantitative analyses.

The development of the Italian Toolkit for the CHAT system was guided by explicit inclusion and exclusion criteria applied before any evaluation or adaptation. Inclusion criteria were defined to 1) ensure relevance to adult clinical populations: only symbols applicable to speech transcripts and error coding of adult speakers were considered; 2) include symbols capturing linguistic phenomena associated with neurologically based language disorders, such as neurodegenerative conditions (e.g., mild cognitive impairment (MCI), primary progressive aphasia (PPA), Parkinson’s disease (PD), Alzheimer’s disease (AD), and other dementias) and post-stroke aphasia.

Symbols referring to child language, typical language development, or linguistic phenomena not relevant to adult neurological language disorders were excluded from consideration. These comprised conversational analysis notation (e.g., tone direction markers) and the subset of symbols in the original CHAT taxonomy dedicated to stuttering-like disfluencies.

### Design of the study

**Fig. 1:**
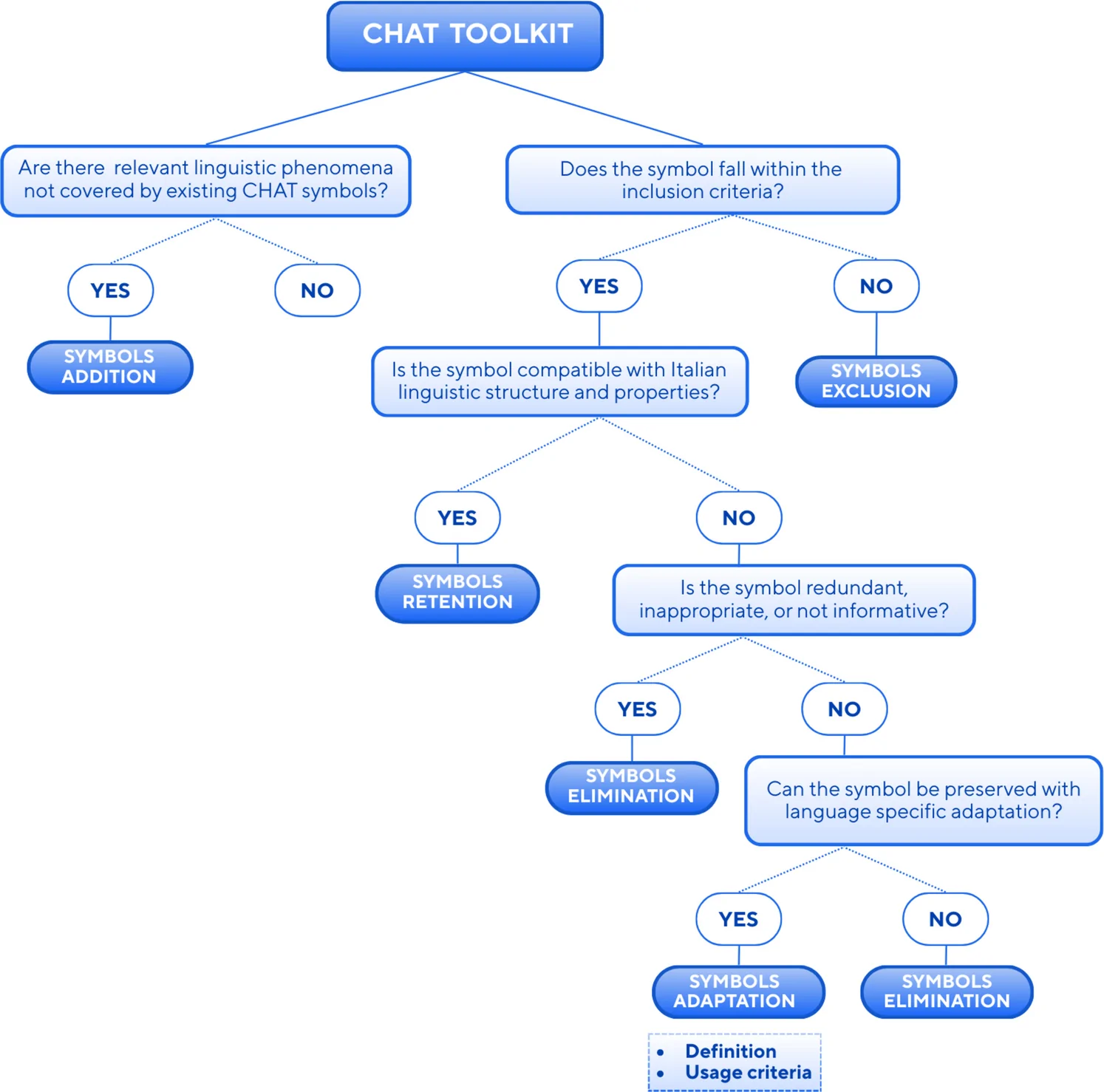
Decision tree guiding symbol selection and adaptation for the Italian CHAT Toolkit outlining the stepwise evaluation process applied to each CHAT symbol during the Italian adaptation, leading to decisions of retention, elimination, adaptation, or addition

The study followed a structured, stepwise decision-making framework for selecting and adapting CHAT symbols to Italian, formalized in the decision tree presented in Fig. 1. Each symbol described in CHAT Transcription format (v. September 1, 2022) was evaluated against a set of predefined decision criteria that guided its retention, elimination, adaptation, or, when necessary, the introduction of a new symbol.

Eligible symbols from the original CHAT manual underwent a symbol-by-symbol evaluation phase. Symbols were *retained* when their original definition and usage were fully compatible with Italian linguistic properties.

Symbols were *eliminated* when they were incompatible with Italian linguistic structure and properties, redundant, or not informative for the aims of the study. For example, the secondary stress marker was removed, as Italian is a syllable-timed language and does not systematically encode secondary stress at the lexical level. Likewise, the multiple repetition marker was excluded, as repetition phenomena were already sufficiently captured by the existing repetition symbol, rendering the additional specification redundant.

Symbols were *adapted* when the original notation was preserved but required language-specific modifications. Adaptation involved either modifying the definition and restricting the range of available subtypes or redefining applicational criteria to reflect Italian linguistic rules while maintaining consistency with the CHAT framework. For instance, phonological error coding was simplified to two operational categories (e.g., [* p] and [* p:n]), reducing the original subtype organization while preserving the distinction between recognizable phonological errors and phonological neologisms. Similarly, the grammatical error marker ([+ gram]) was retained but restricted to instances in which Italian grammatical well-formedness was compromised, given the permissibility of null subjects and ellipsis in Italian (Russo et al., 2012). Moreover, new symbols were introduced when recurrent linguistic phenomena observed in adult-acquired neurological language could not be adequately represented using the available CHAT notation. In particular, an articulatory distortion marker ([* w]) was added to capture phonetic-level distortions associated with motor speech impairments (e.g., apraxia of speech or dysarthria) (Duffy, 2006), which were highly prevalent in the analyzed clinical sample and not explicitly encoded within the original system.

All the symbols were subsequently organized into a hierarchical structure (i.e., word level and sentence level; transcription first and error coding second), maintaining the original CHAT manual’s distinction between transcription and error-coding symbols. Within each section, symbols were further classified by linguistic units (utterance level vs. word level), providing an operational structure suitable for real-time transcription. Error-coding symbols were additionally grouped into four linguistic domains (phonetic–phonological, lexical–semantic, morphosyntactic, and pragmatic/discourse), in line with the classification framework proposed by Boschi et al. (2017).

### Clinical application and cross-linguistic comparison

To demonstrate the clinical and discriminative utility of the Italian CHAT adaptation, a connected speech sample produced by a patient with the non-fluent variant of primary progressive aphasia (nfvPPA) was used as an illustrative example.

Briefly, nfvPPA is a neurodegenerative condition characterized by effortful speech production, articulatory difficulties, and agrammatism; preserved single-word comprehension is often associated with difficulties in understanding and producing syntactically complex sentences (Gorno-Tempini et al., 2011). The speech of nfvPPA often appears simplified, telegraphic, and, in some cases, unintelligible (Rezaii et al., 2024). Thus, this case was selected because the speech and language characteristics of nfvPPA capture several of the most relevant phenomena that required adaptation or modification when transferring the CHAT conventions from English to Italian. G.B. is a right-handed 64-year-old female with sixteen years of formal education and was referred to our Laboratory of Neuropsychology after a 2-year history of progressive language deficits. During her first visit to our lab, G.B. reported articulation difficulties and effortful production, and she produced only a few long sentences. She experienced difficulties with writing, including spelling errors, and produced occasional morphological and phonological errors in oral production. Her CDR score was 0.5, and her Progressive Aphasia Severity Scale (PASS) (Sapolsky et al., 2014) was 3.5, indicative of mild difficulties in articulation and in fluency.

G.B.’s connected speech was elicited using the “Summer Time” description task of the Screening for Aphasia in Neurodegeneration (SAND) (Battista et al., 2018). A comparison was performed between the connected speech sample transcribed in Italian using the adapted toolkit and its English translation, transcribed according to the original CHAT conventions. This clinical case is presented for illustrative purposes only, as a descriptive demonstration of the proposed Italian conventions compared to the English conventions, and does not constitute an inferential analysis. Transcription and coding were performed by the first author and independently checked by the third author; in cases of disagreement, the corresponding author was consulted to reach consensus.

## Results

### Transcription symbols

**Fig. 2:**
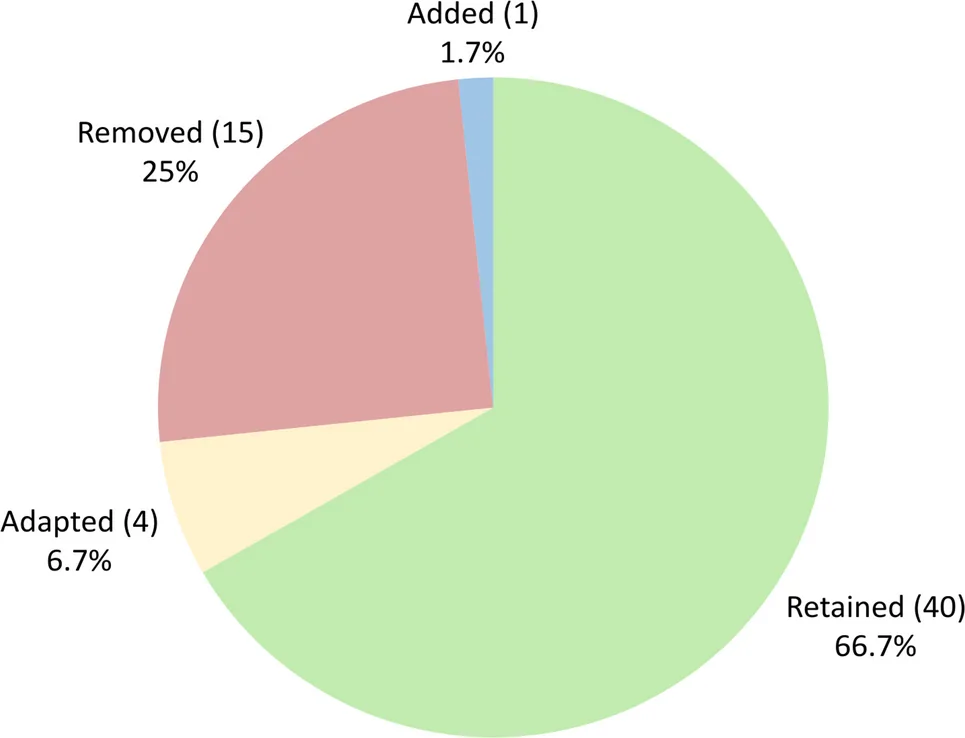
Distribution of transcription symbols in the Italian adaptation of the CHAT manual. The pie chart represents the quantitative results of the Italian adaptation process, specifically for transcription symbols within the CHAT manual. The distribution includes retained original symbols (*n* = 40), symbols adapted for the Italian language (*n* = 4), newly added symbols (*n* = 1), and removed symbols (*n* = 4). Percentages reflect the proportion of each category relative to the total set of symbols (*n* = 59)

A total of 59 transcription symbols were evaluated. The selection process (see the decision process, Fig. 2), resulted in 40 symbols being *retained* without modification, 15 *removed*, four *adapted*, and one newly *added*.

Detailed examples of the use in Italian of the transcription symbols that were adapted or newly introduced are provided in Supplementary Table 1. The complete inventory of transcription and error-coding symbols forming the final Italian-adapted toolkit is reported in Appendix Tables 1 and 2. Appendix 1 constitutes the operational guideline of the Italian CHAT Toolkit and is intended as a downloadable reference document. Rather than a standalone application, it provides the complete set of adapted conventions and instructions for integrating them into the standard CHAT/CLAN transcription and coding procedure for Italian samples.

### Utterance level

At the utterance level, six symbols were eliminated. The transcription break (+.) was removed following updated CHAT recommendations, as overlaps are more accurately represented via the &*ID:word notation. Standard punctuation markers such as the comma (,), semicolon (;), and colon (:), were excluded because they do not encode structural syntactic units, define units of analysis, nor influence automated CLAN outputs (i.e., mean utterance length). Furthermore, the multiple-repetition marker ([x N]) was deemed redundant, and the unclear retracing type ([/?]) was removed, as it is only necessary for SALT–CHAT conversion.

Additionally, the clause delimiter ([^c]) was adapted. The original notation was retained, but its operational use was refined. Beyond the original CHAT definition, this clause delimiter was applied specifically to tag subordinate clauses, providing a direct metric for Italian syntactic complexity.

### Word level

At the word level, four symbols were eliminated. Secondary stress and lengthened-syllable markers were removed because of the syllable-timed nature of Italian and the influence of non-pathological dialectal variation in vowel lengthening, which could confound clinical interpretation. The marker for untranscribed material (www) was also excluded to reduce transcription density without compromising analytical value. Moreover, the phonological string marker (yyy) was removed because it requires the inclusion of a phonetic (%pho) tier. All identifiable non-lexical productions are instead captured under the unified non-word category (& ~).

Furthermore, two symbols underwent adaptation. Specifically, the incomplete-word format (text) was adapted to reflect Italian abbreviation patterns, while the omitted-word marker (0word) was restricted to contexts in which omission violates Italian grammatical constraints. In English, the omission of subjects, auxiliaries, or functional particles often constitutes a clear grammatical violation; by contrast, Italian permits a wider range of null elements within context-dependent structures.

Finally, a new word fragment marker (&/) was introduced to capture partial word productions that are abandoned without reformulation or a precisely recognizable lexical target; this phenomenon, frequently observed in adult neurological speech, was not fully representable using the existing CHAT notation.

Event-related symbols (including local and scoped events) were reviewed and categorized separately, as they primarily capture contextual or interactional phenomena rather than the internal structural or lexical features of the utterance. Their full specification is provided in Appendix Table 1.

### Error-coding symbols

**Fig. 3:**
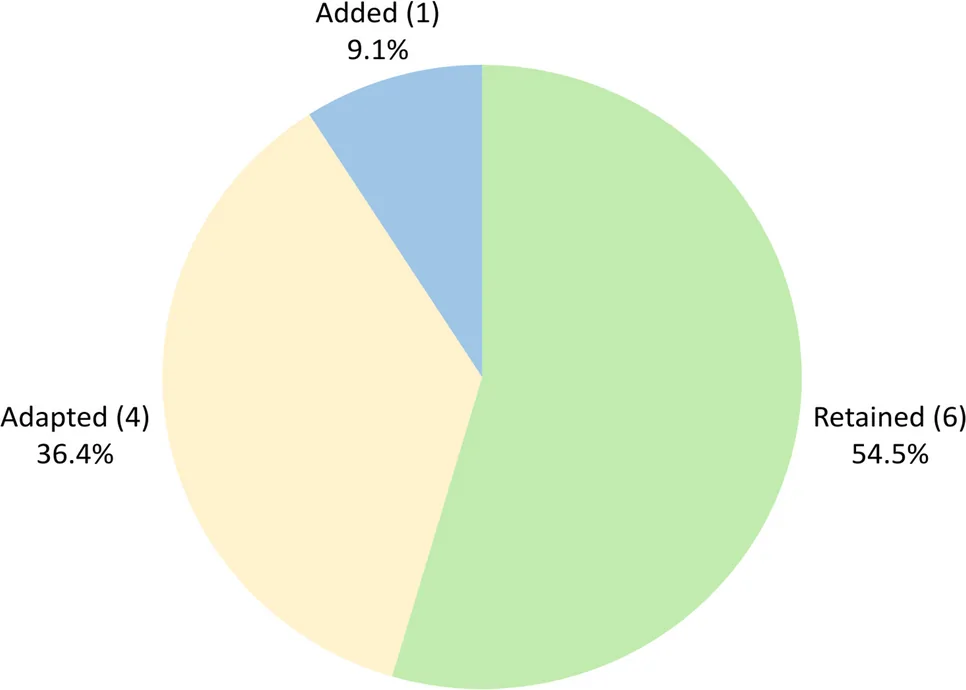
Distribution of error coding symbols in the Italian adaptation of the CHAT manual. The pie chart represents the quantitative results of the Italian adaptation process, specifically for error coding symbols within the CHAT manual. The distribution includes retained original symbols (*n* = 6), symbols adapted for the Italian language (*n* = 4), and newly added symbols (*n* = 1). Percentages reflect the proportion of each category relative to the total set of error coding symbols (*n* = 11)

A total of 11 error-coding symbols were considered for inclusion in the toolkit. Of these, six were retained, four adapted, and one newly introduced (Fig. 3). No error-coding symbols were eliminated. As reflected in Appendix Table 2, error-coding symbols were organized into utterance/discourse-level and word-level categories and further classified by linguistic domain (Boschi et al., 2017). Worked examples of the adapted and newly introduced error-coding symbols are provided in Supplementary Table 2.

Across the different levels, changes were concentrated in the phonetic–phonological and morphosyntactic linguistic levels. Specifically, at the utterance level, one phonetic–phonological and one morphosyntactic symbol were modified. At the word level, the phonetic–phonological domain accounted for one adaptation and one newly introduced symbol, while the morphosyntactic domain involved one adaptation.

### Utterance/discourse level

At the utterance level, the fluency alteration marker ([*], [* d]) was adapted to focus exclusively on phenomena (e.g., empty or filled pauses, fillers, false starts, retracing) that affect the temporal and structural organization of the utterance, while excluding stuttering-like disfluencies.

Lexical–semantic and discourse-level codes were retained without modification. Lexical–semantic and discourse codes, including circumlocution ([+ cir]), perseveration ([+ per]), jargon ([+ jar]), and empty speech ([+ es]), were retained without modification. The grammatical error marker ([+ gram]) was adapted to account for Italian syntactic permissiveness, ensuring it is applied only when well-formedness is compromised according to Italian-specific morphosyntactic rules rather than English structural expectations.

### Word level

Word-level error-coding was refined to enhance clinical reliability. Phonological coding was streamlined from the extensive original CHAT taxonomy into two primary operational categories: [* p] representing recognizable phonological errors (e.g., omissions, substitutions, insertions, or metatheses), and [* p:n], representing phonological neologisms. Notably, the latter may co-occur with non-word symbols at the transcription level to document cases of complete target loss.

Morphological coding ([* m:x]) was similarly restricted to two subtypes to better reflect the Italian inflectional system: [* m:a] for morphological agreement errors (gender, number, or person) and [* m:v] for verbal morphology errors (tense, mood, or irregular inflections).

Finally, a new word-level symbol, [* w], was introduced to specifically code articulatory distortions. This marker captures phonetic distortions affecting sound realization despite correct phonological sequencing, providing a vital metric for motor speech disorders such as apraxia of speech or dysarthria. Its identification is further supported by the occurrence of broken words, phonological fragments, or distorted phonetic realizations.

### Clinical application and cross-linguistic comparison

**Fig. 4:**
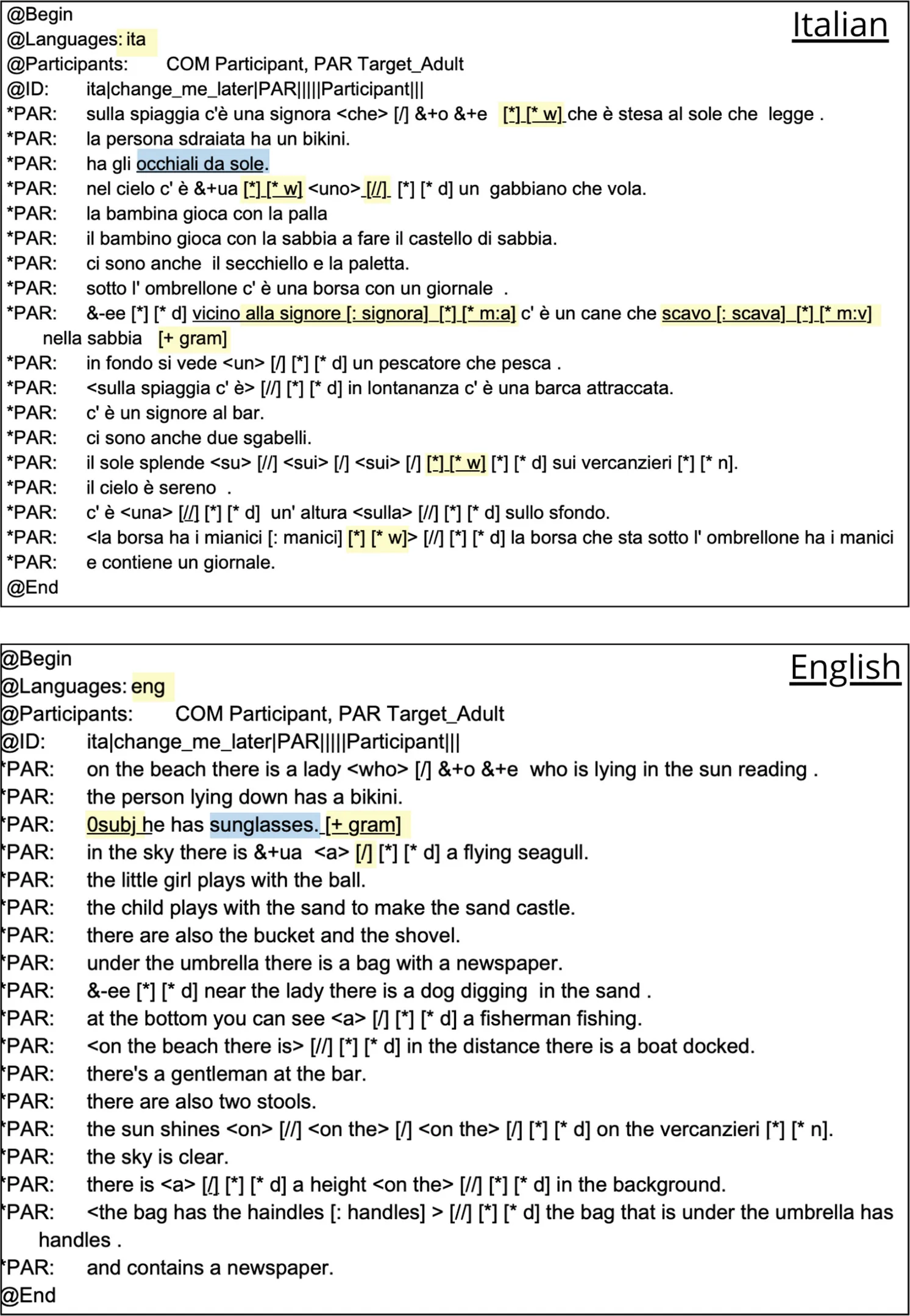
Cross-linguistic comparison of Italian and English CHAT transcriptions of the same connected speech sample, produced using the Italian-adapted CHAT Toolkit and the original CHAT conventions, respectively

Figure 4 displays a comparison between the G.B. connected speech sample, transcribed in Italian using the adapted toolkit, and its English translation, transcribed according to the original CHAT conventions.

Several language-dependent differences emerge between the two representations (highlighted in yellow in the figure). First, the Italian transcript illustrates phenomena motivating the introduction of the articulatory distortion marker ([* w]). Its occurrence in association with repeated phonological fragments and retracing sequences reflects phonetic struggle consistent with motor-speech impairment. Although such behaviors can be represented structurally in standard CHAT transcription, they were not previously directly quantifiable at the error-coding level. Additional divergences emerge within the morphosyntactic domain. In Italian, subject omission (*ha gli occhiali da sole—has sunglasses*) constitutes a grammatically licensed structure and therefore does not require grammatical error marking. In contrast, the English transcription requires the explicit insertion of a null subject (0subj) and the assignment of the [+ gram] code, reflecting language-specific syntactic constraints rather than a pathological impairment.

Finally, although prosodic annotation within words was not implemented in the present transcription, the compound noun highlighted in blue (*occhiali da sole* vs. *sunglasses*) exemplifies a cross-linguistic difference that would emerge under prosodic analysis. English compound nouns typically rely on hierarchical primary and secondary stress patterns (e.g., *‘sun,glasses*), whereas Italian lexical items generally exhibit a single primary stress (*occhi’ali da’ sole*).

## Discussion

This work developed a structured Toolkit for adapting speech and language transcription and coding conventions from English to Italian for adult speakers with neurological disorders. The extension builds on the CHAT conventions developed within the TalkBank framework and follows its guiding principles of clarity, readability, and data-entry efficiency (MacWhinney, 2000), while remaining sensitive to the structural properties of Italian.

The original commands and coding options were systematically reviewed and retained, modified, excluded, or expanded as necessary, resulting in a coherent, operational manual applicable in both Italian research and clinical contexts. In the original English version, the phonetic and articulatory domain was not fully captured, and the available symbols were therefore insufficient to fully characterize the speech of patients with motor speech disorders. These difficulties, however, represent a key aspect of speech production and are essential for supporting the differential diagnostic process (i.e., dysarthria from apraxia of speech from phonological difficulties). Accordingly, additional symbols were introduced to better capture the phonetic-phonological and motor speech deficits observed in individuals with aphasia, providing a more complete representation of disfluency patterns and connected speech in adult neurological populations (Cordella et al., 2024; Mueller et al., 2018; Poole et al., 2017; Vonk et al., 2025). Moreover, the English original version automatically accounts for syntactic complexity, while this function was not available for Italian. Thus, we suggest that the use of [^c] symbol can be helpful to capture those sentences that carry syntactic complexity in Italian. This is relevant to better describe the agrammatic production of aphasic patients, which otherwise remains underexplored and may play a critical role in defining agrammatism (Bates et al., 1987). Indeed, such modifications reflect important morphosyntactic characteristics of Italian, including the definition and identification of agrammatic utterances and the characterization of morphological errors. Another relevant aspect that we introduced in the Italian Toolkit concerns the disfluency &/symbol, which denotes abandoned words that cannot be associated with an identifiable target. This choice was driven by the need to accurately reflect the lexical retrieval difficulties, as it is the most frequent aphasic disorder (Akhmadullina et al., 2023; Lambon Ralph et al., 2010; Reilly et al., 2011).

Beyond its Italian adaptation, this toolkit offers clear guidelines that can inform similar efforts in other languages and clinical populations, providing a reference for addressing adaptation challenges and implementing new symbols. This work is particularly relevant because most of the world’s population does not speak English, yet research and clinical tools remain overwhelmingly Anglophone. Because each language has unique structural features, directly using English-language instruments often fails for diagnosis, treatment, and cross-cultural comparison (Alladi & Hachinski, 2018). In a recent study, we compared speech and language production in healthy speakers of English, Italian, Cantonese, and Mandarin, highlighting significant cross-linguistic differences, particularly in the use of function words and in the phonological domain, which reflect language-specific morphosyntactic rules. The findings further support the notion that linguistic diversity can shape the manifestation of language processing patterns (Santi et al., under review). Cross-linguistic research depends on shared procedures to ensure comparability and support cumulative analyses, yet practical guidance on adapting existing annotation systems or tasks to structurally different languages remains limited (Kristjansson et al., 2003). One of the main methodological challenges is finding an appropriate balance between standardization and linguistic specificity: direct transfer across languages risks overlooking language-dependent structures, whereas extensive language-specific modifications may reduce compatibility. The approach proposed here aims to preserve the conceptual organization and operational principles of the original TalkBank conventions while introducing targeted adjustments in its use, motivated by the linguistic structure of Italian. Maintaining this balance is crucial if standardized annotation systems are to remain both comparable across languages and linguistically appropriate.

Furthermore, the toolkit was conceived as a practical and accessible resource, with particular attention to clarity of procedures and their applicability in real-world scenarios (Aresta et al., 2025). Detailed coding systems offer clear analytical advantages, but their complexity can limit their use outside specialized research environments. Emphasizing transparent coding rules and streamlined data entry was therefore a central goal of the adaptation, aimed at facilitating the routine use of standardized speech and language annotation in clinical practice and supporting the development of comparable datasets across research groups (Lanzi et al., 2023). In order to demonstrate the practical utility and applicability of the toolkit in the clinical setting, we described an illustrative clinical example that shows how properties of the target language shape transcription and coding decisions in practice, and why cross-linguistic adaptation is necessary when evaluating language production (Blasi et al., 2022; Evans & Levinson, 2009). In fact, the toolkit allows users to select symbols and coding options guided by specific clinical or research interests, rather than requiring exhaustive annotation. This flexible structure enables users to select among the Italian-relevant features those most appropriate for exploring or characterizing particular aspects of language production, whether at the sentence or word level or across different linguistic domains (i.e., phonetic–phonological, lexical–semantic, morphosyntactic, and discourse domains).

## Limitations and conclusion

Some limitations should be considered. The present adaptation was developed specifically for adult speakers with neurological disorders and does not directly address transcription needs in other populations, such as developmental groups, where additional modifications would likely be required. In addition, despite efforts to simplify the conventions, coding remains time-consuming and may constrain large-scale implementation. Future work should therefore explore closer integration between standardized coding schemes (Liu et al., 2023) and automated transcription approaches (e.g., Whisper (Radford et al., 2022)). Applying the proposed conventions to a wider range of datasets and clinical populations will also be important for assessing their robustness and generalizability.

Taken together, this toolkit provides a practical resource for characterizing Italian-language. It also constitutes a transparent model for cross-linguistic adaptation of annotation systems. Developing shared but adaptable annotation practices is an important step toward more cumulative and comparable cross-linguistic research on language impairment. A natural extension of this work concerns the morphosyntactic annotation layer: because the %mor tier is adapted not only on linguistic grounds but also according to the specific research hypothesis and clinical question, its development for Italian represents a distinct line of work, to be designed jointly around the properties of the language and the aims of the user. At the same time, the present work underscores the importance of adapting language coding and analysis tools to specific linguistic and clinical contexts to support valid, clinically meaningful assessment of adult neurological speech and language. By providing a linguistically grounded set of conventions for Italian, the toolkit aims to enhance the applicability and clinical relevance of standardized speech analysis in Italian-speaking adults.

## Electronic supplementary material

Below is the link to the electronic supplementary material.

Supplementary file1 (PDF 131 kb)

## Data Availability

All materials are available in Appendix 1. Appendix 1 serves as the downloadable operational guideline; the toolkit is not distributed as a standalone software resource.

## Appendix 1

**Table 1 Transcription Guidelines for Italian Symbols. Summary of retained, adapted or removed symbols for transcription:** This is mandatory

**Transcription symbols related to the utterance-level unit**					
**Macro-category**	**Symbol**	**Original English CHAT definition**	**Decision**	**Adaptation/Use in Italian**	**Notes**
Syntactic structure and delimitation of the utterance		Period. Marks the end of a declarative utterance	**Retained**	—	—
	**?**	Question mark. Marks the end of an interrogative utterance	**Retained**	—	—
	**!**	Exclamation point. Marks imperatives or utterances produced with emphasis	**Retained**	—	—
	** + …** ** +..?**	Trailing off/trailing off question. The utterance remains incomplete because the speaker stops due to distraction or loss of planning, not due to interruption by another speaker. The utterance fragment produced meets the criteria for a well-formed utterance	**Retained**	—	Utterances marked with trailing off are included in MLU counts
	** +!?**	Question with exclamation. Interrogative utterance produced with strong emphasis; syntactically an interrogative, prosodically exclamative	**Retained**	—	—
	** +/** ** +/?**	Interruption by another speaker. The speaker cannot complete the utterance due to interruption. When resuming, the new utterance begins with +,; if the interrupted utterance was a question, +/? is used	**Retained**	—	Utterance fragments produced by the same speaker are treated as a single utterance in MLU counts only if (+/) and (+,) are used in combination
	** +//** ** +//?**	Self-interruption. The speaker interrupts themselves and then resumes speaking. The resumed utterance begins with +,	**Retained**	—	—
	**[^c]**	Clause delimiter. Marks the end of a clause within a complex sentence	**Adapted**	Used in two ways: (1) following the original CHAT definition when clauses are the unit of analysis; (2) applied only at the end of subordinate clauses to quantify syntactic complexity at the utterance level	—
	** + **	Transcription break. Marks overlap between speakers without creating a new utterance	**Removed**	Replaced by &*ID:word	Considered redundant according to updated CHAT recommendations
	**&*ID:word**	Overlap/interposed word. A second speaker inserts a single word (e.g., “mh”, “yeah”) without taking the floor	**Retained**	—	
Connection and integration of the utterance	** + ^**	Quick uptake. Marks immediate, impulsive responses and tight turn-taking	**Retained**	—	—
	** +,**	Self-completion. Used when a speaker resumes an utterance after interruption or self-interruption	**Retained**	—	—
	** + + **	Other-completion. Another speaker completes the utterance initiated by the first speaker	**Retained**	—	—
	** + "/;** ** + ";** **" "**	Quotation. Used to introduce direct speech within narratives. Long quotations are placed on a new line preceded by + "; short quotations remain on the same line and are enclosed in quotation marks	**Retained**	—	—
Internal structure/organization of the utterance	**‡**	Initial satellite. Marks vocatives or peripheral material at the beginning of the utterance, excluded from morphosyntactic analysis	**Retained**	—	—
	**„**	Final satellite. Marks utterance-final elements (e.g., question tags, final particles)	**Retained**	—	—
	**,**	Comma. Non-structural separator indicating minor intonational breaks	**Removed**	—	These symbols do not encode syntactic structure, do not define units of analysis, and do not affect automated CLAN outputs (e.g., MLU, MOR)
	**;**	Semicolon. Used only in highly formal transcriptions; in CA marks light final intonation drop	**Removed**	—	
	**:**	Colon. Non-formal separator; within words marks phonetic lengthening	**Removed**	—	
	**[/]**	Repetition. Repetition of words, fragments, or entire utterances	**Retained**		Repeated multi-word material is enclosed in < … >
	**[x N]**	Multiple repetitions	**Removed**	—	Considered redundant
	**[//]**	Retracing. Reformulation preserving the same idea with local changes	**Retained**	—	Associated with self-correction, motor planning, or lexical access difficulties
	**[///]**	Reformulation. Complete reformulation of the message without shared material	**Retained**	—	Reflects re-planning and loss of discourse thread
	**[/-]**	False start without retracing. Abandoned utterance not repeated or reformulated. The produced segment does not meet utterance criteria	**Retained**	Used only when the produced segment does not meet utterance criteria	Material enclosed in < … > is excluded from MLU
	**[/?]**	Unclear retracing type. Used only for SALT–CHAT conversion	**Removed**	—	—
	**[e], [+ exc]**	Excluded material. Marks material excluded from analysis ([e] partial; [+ exc] entire utterance)	**Retained**	—	—
**Transcription** **symbols related to the word- level unit**					
**Macro-category**	**Symbol**	**Original English CHAT definition**	**Decision**	**Adaptation/Use in Italian**	**Notes**
Prosody within words	**'**	Primary stress. Marks the stressed syllable	**Retained**	—	
	**,**	Secondary stress	**Removed**	—	Not applicable to Italian (syllable-timed language)
	**:**	Lengthened syllable	**Removed**	—	Highly transcriber-dependent dialectal variation in Italian
	**^**	Pause between syllables. Articulatory pause within a word	**Retained**	—	Used as an indicator of phonetic struggle or groping
Status and realization of the word	**@ (@d, @s:***)**	Special form marker. Non-standard or non-dictionary word forms	**Retained**	@d = regional/dialectal lexical form (e.g., sedia@d); @s:*** = form from another language (*** = language code, e.g., @s:eng). Subcategories of the retained @ marker	Treated as full words and included in length-based measures (e.g., MLU); by default not assigned a part of speech by MOR. For morphosyntactic analysis, the recognized standard target form can be given in the replacement brackets [: XXX] (e.g., home@s:eng [: casa]), so that the form is analyzed on the %mor line
	**xxx**	Unintelligible word	**Retained**	—	Ignored in MLU calculations
	**yyy**	Phonologically coded material with no orthographic correspondence	**Retained**	—	Requires a %pho tier
	**www**	Untranscribed material on the main line	**Removed**	—	Increases transcription and analysis complexity
	**& + **	Phonological fragment not completed and not corresponding to the following lemma	**Retained**	—	—
	**&-**	Filler. Planning and discourse-maintenance signal	**Retained**	—	—
	**& ~ **	Non-word. "This symbol is used for nonwords that are not fillers or phonological fragments and which are not characterized by any special form marker."	**Retained**	—	—
	**text(text)text**	Incomplete word. Partial production with clear intended meaning	**Adapted**	Applied according to Italian abbreviation conventions	—
	**0word (0det, 0prep, 0aux, 0 cl, 0comp, etc.)**	Omitted word	**Adapted**	Used only when omission violates Italian grammatical rules. The "0" prefix applies to any obligatory grammatical class (determiner, preposition, complementizer, auxiliary, pronoun, clitic); the null subject is licensed in Italian and is left unmarked	0v → + gram; 0n → semantic error
	**&/**	Word fragment. Partial word production abandoned without reformulation and with no identifiable target	**Added**	—	—
**Transcription symbols related to the event- related unit**					
**Macro-category**	**Symbol**	**Original English CHAT definition**	**Decision**	**Adaptation/Use in Italian**	**Notes**
Local events	**& = **	Simple local event (e.g., cough, laugh, gesture)	**Retained**	—	—
	**[^ testo]**	Complex local event localized at a specific point	**Retained**	—	—
	**&{l = * … &}l = ***	Long vocal event	**Retained**	—	—
	**&{n = * … &}n = ***	Long non-vocal event	**Retained**	—	
	**&*ID:word**	Interposed word during overlap	**Retained**	—	Used instead of +
	**(.)** **(..)** **(…)**	Silent pauses (short, medium, long)	**Adapted**	Temporal duration not specified	Pauses may be an indicator of anomia
	**[>] [<] + < **	Overlap markers (follow, precede, lazy overlap)	**Removed**	—	—
Explanation and alternatives (scoped events)	**[= text]**	Explanation of the referenced material	**Retained**	—	—
	**[: text]**	Replacement. Standardized form used for MOR analysis	**Retained**	—	—
	**[:: text]**	Replacement of real words	**Removed**	—	—
	**[=? text]**	Alternative transcription	**Removed**	—	—
	**[% comment]**	Comment on the main line	**Removed**	—	—
	**[?]**	Best guess	**Removed**	—	—

**Table 2 Error-Coding Guidelines for Italian Symbols. Summary of retained and adapted utterance and discourse-level errors coding:** This is mandatory

**Utterance and Discourse level error-coding**					
**Linguistic domain**	**Symbol**	**Original English CHAT definition**	**Decision**	**Adaptation/Use in Italian**	**Notes**
Phonetic phonological	**[*] [* d]**	Fluency alteration. Disruptions affecting the temporal and structural organization of the utterance	**Adapted**	Pauses, fillers, false starts, retracing, and interruptions impacting utterance fluency	—
Lexical—Semanthic	**[+ cir]**	Circumlocution. A compensatory strategy used when lexical retrieval fails, resulting in the production of multi-word descriptions rather than the intended lexical item	**Retained**	Multi-word strategy used to compensate for failure to retrieve a target lexical item or access a semantic concept; treated as an utterance/discourse-level manifestation of a word-level lexical access deficit	—
Morphosyntactic	**[+ gram]**	Agrammatic or paragrammatic utterances, including omission of function words, incorrect word order, or malformed syntactic structures	**Adapted***	We must considerItalian-specific syntactic permissiveness Italian allows null subjects and ellipsis; [+ gram] is used only when grammatical well-formedness is compromised	—
Pragmatics/discourse organization	**[+ per]**	Perseveration. Inappropriate persistence or repetition of prior lexical/discourse material impairing discourse progression	**Retained**	—	—
	**[+ jar]**	Jargon. Fluent output with severely reduced intelligibility and/or semantic transparency and impaired message-level coherence	**Retained**	—	—
	**[+ es]**	Empty speech. Grammatically correct output with low informational content and reduced referential specificity (vague lexical items)	**Retained**	—	—
**Words level error-coding**					
**Linguistic domain**	**Symbol**	**Original English CHAT definition**	**Decision**	**Adaptation/Use in Italian**	**Notes**
Phonetic– phonological	**[* p]** **p:w word,** **p:n non-word,** **p:m metathesis,**	Phonological error. Involve substitution, omission, addition, or metathesis of phonemes, preserving part of the syllabic structure. For monosyllabic words, at least 2 of 3 syllabic components (onset–nucleus–coda) must match; for multisyllabic words, all but one syllable must match	**Adapted**	[* p]: Omission, substitution, insertion, or metathesis of phonemes, resulting in an incorrect but still recognizable target word [* p:n]; Phonological neologism; phoneme-level distortion resulting in loss of target word recognizability	Cases coded as phonological neologisms ([p:n]) typically co-occur with non-word symbols at the transcription level, reflecting the absence of a recognizable lexical target
	**[* w]**	—	**Added**	Articulatory distortion: production of sounds not belonging to the phonetic inventory of the language, despite correct phonological sequencing.Distorted phonetic realization due to motor speech impairment (e.g., apraxia of speech, dysarthria)	Identified indirectly through co-occurrence with broken words (^), phonological fragments (& +), repeated fragments with retracing, or distorted phonemes
Lexical–semantic	**[* s]**	Semantic errors. Semantic paraphasia (related or generalized substitution) and anomia (failure to retrieve the target word 0n or 0v)	**Retained**	—	Semantic paraphasia (related or generalized substitution) and anomia (failure to retrieve the target word)
	**[* n]**	Neologism. Lexically structured but non-existing word replacing a target	**Retained**	—	—
Morphosyntactic	**[* m: x] (subtypes: m:a, m:v, m:s:CAT)** **x specify the morphological error subtype**	Morphological errors. Involve the incorrect use, omission, or substitution of inflectional or derivational morphemes within a word, including errors of agreement, tense, number, person, gender, or other grammatical features	**Adapted**	[*m:a] Incorrect morphological agreement involving number, gender, or person features on a single lexical item [*m:v] Incorrect verbal inflection involving tense, mood, or irregular verb forms [* m:s:CAT] Substitution of an incorrect free functor, where CAT is the grammatical class involved (e.g., prep, art, cl). Auxiliary selection is coded as m:v, since the auxiliary is part of the verbal compound. When such a substitution compromises well-formedness it is additionally captured by [+ gram]	—
