# Supplementary material for: Extending standardized speech analysis across languages: An Italian toolkit for transcription and coding of neurological speech
Source: Behav Res Methods. 2026 Sep 14;58(10):292. doi: 10.3758/s13428-026-03175-x (PMC13574730; doi:10.3758/s13428-026-03175-x)
Supplement: Supplementary file 1 [file 13428_2026_3175_MOESM1_ESM.pdf]

Supplementary Material

Supplementary Table 1. Transcription symbols
*Illustrative examples of the transcription symbols adapted or newly introduced in the Italian CHAT Toolkit. CHAT-formatted productions are shown on the main (*PAR) tier, with English glosses in italics where relevant.*

Symbol	Type	Example (CHAT)	Explanation
[^c]	**Adapted**	*PAR: la donna dice che è stanca [^c] perché ha lavorato troppo [^c] .<br>*'the woman says that she is tired because she worked too much'*	Clause delimiter. In the Italian-specific use, [^c] is placed only at the end of subordinate clauses to quantify syntactic complexity; the main clause (*la donna dice*) is left unmarked.
text(text)text	**Adapted**	*PAR: prendo la bici(cletta) .<br>*'I take the bike(cycle)'*	Incomplete word. Only *bici* is produced; the unrealized portion is given in parentheses, following Italian abbreviation patterns.
0word (0det, 0prep, 0aux, 0cl, 0comp, etc.)	**Adapted**	*PAR: 0det bambino mangia 0det mela .<br>*PAR: vado 0prep scuola .<br>*PAR: penso 0comp viene .<br>*PAR: Maria 0cl vede .	Omitted word. The "0" prefix applies to any obligatory grammatical class; marked only when the omission compromises Italian well-formedness. The null subject is licensed and therefore not marked.
(.) (..) (...)	**Adapted**	*PAR: la signora prende il (.) ombrello .<br>*'the lady takes the (.) umbrella'*	Silent pauses, coded Coded without specifying temporal duration to avoid operator-dependent bias.
&/	**Added**	*PAR: lei vuole gio&/ a palla .<br>*'she wants pla&/ with the ball'*	Word fragment. A partial production abandoned, not reformulated, with no identifiable lexical target (distinct from &+, a phonological fragment related to the following lemma).

For Review Only

Supplementary Table 2. Error-coding symbols
*Illustrative examples of error-coding symbols adapted or newly introduced in the Italian CHAT Toolkit. CHAT-formatted productions are shown on the main (*PAR) tier, with English glosses in italics where relevant.*

Symbol	Type	Example (CHAT)	Explanation
[*] / [* d]	**Adapted**	*PAR: e poi &-ehm la signora (.) apre [/] apre la porta [* d] . *'and then ehm the lady opens opens the door'*	Fluency alteration. A filler, a pause, and a retracing disrupt the temporal organization of the utterance; one [* d] per disfluent segment; stuttering-like disfluencies are excluded.
[* p] / [* p:n]	**Adapted**	*PAR: bicchiele [: bicchiere] [* p] . *PAR: &~tellofo [: telefono] [* p:n] .	[* p]: phonological error with a recognizable target. [* p:n]: phonological neologism (target no longer recoverable), co-occurring with the non-word symbol &~.
[* m:a] / [* m:v] / [* m:s:CAT]	**Adapted**	*PAR: la macchina rosso [: rossa] [* m:a] . *PAR: ieri lui ha prenduto [: preso] [* m:v] . *PAR: io ho [: sono] [* m:v] andato . *PAR: vado in [: a] [* m:s:prep] Roma . *PAR: il [: un] [* m:s:art] bambino gioca . *PAR: gli [: le] [* m:s:cl] parlo .	**Morphological/morphosyntactic errors.** m:a = agreement (feature mismatch: gender, number, person). m:v = verbal morphology (tense, mood, irregular forms, and auxiliary selection in compound tenses, since the auxiliary is part of the verbal compound). m:s:CAT = substitution of an incorrect free functor outside the verbal complex (m = morphosyntactic domain, s = substitution, CAT = grammatical category: prep, art, cl). When such a substitution compromises well-formedness, it is additionally captured by [+ gram].
[* w]	**Added**	*PAR: ca^ca^cane [* w] . *'d-d-dog'*	Articulatory distortion. A distorted phonetic realization with intra-syllabic pauses (^), typical of motor speech impairment (apraxia of speech, dysarthria); distinguishing it from stuttering-like syllable repetition is left to clinical judgment.

For Review Only
